# Case report: Treatment response of NF-1-associated bladder ganglioneuroma to trametinib

**DOI:** 10.3389/fonc.2024.1433073

**Published:** 2024-07-12

**Authors:** Marcus C. Y. Chan, Kevin K. F. Fung, Wai-Fu Ng, Ho-Ming Luk, Dennis T. L. Ku, Anthony P. Y. Liu

**Affiliations:** ^1^ Department of Paediatrics and Adolescent Medicine, Queen Mary Hospital, Hong Kong, Hong Kong SAR, China; ^2^ Department of Radiology, Hong Kong Children’s Hospital, Hong Kong, Hong Kong SAR, China; ^3^ Department of Pathology, Hong Kong Children’s Hospital, Hong Kong, Hong Kong SAR, China; ^4^ Clinical Genetics Service Unit, Hong Kong Children’s Hospital, Hong Kong, Hong Kong SAR, China; ^5^ Department of Paediatrics and Adolescent Medicine, Hong Kong Children’s Hospital, Hong Kong, Hong Kong SAR, China; ^6^ Department of Paediatrics and Adolescent Medicine, School of Clinical Medicine, Li Ka Shing Faculty of Medicine, The University of Hong Kong, Hong Kong, Hong Kong SAR, China

**Keywords:** ganglioneuroma, neurofibromatosis type 1 (NF-1), MEK inhibitor, MAPK pathway, targeted therapy

## Abstract

We present the clinical course of a 4-year-old girl with neurofibromatosis type 1-associated, unresectable, symptomatic urinary bladder ganglioneuroma. She was initially trialed on sirolimus without response and subsequently responded to MEK inhibitor trametinib, with improvement clinically and radiographically over 10 months. This report broadens the repertoire of therapeutic strategies for MEK inhibition in diseases related to the MAPK pathway.

## Introduction

Ganglioneuroma is a low-grade tumor arising from neural crest cells, which can be sporadic or associated with cancer predisposition syndromes including neurofibromatosis type 1 (NF-1), multiple endocrine neoplasia type 2B, Cowden syndrome, and Costello syndrome. These tumors may rarely arise from the urinary bladder, presenting with lower tract urinary symptoms and hematuria. To date, only 15 of such cases, including three cases associated with NF-1 were reported, among which most were treated with surgery alone (1–10). We hereby describe a patient with unresectable, NF-1-related urinary bladder ganglioneuroma who showed treatment response to the mitogen-activated protein kinase (MEK) inhibitor trametinib.

## Case presentation

**Figure 1:**
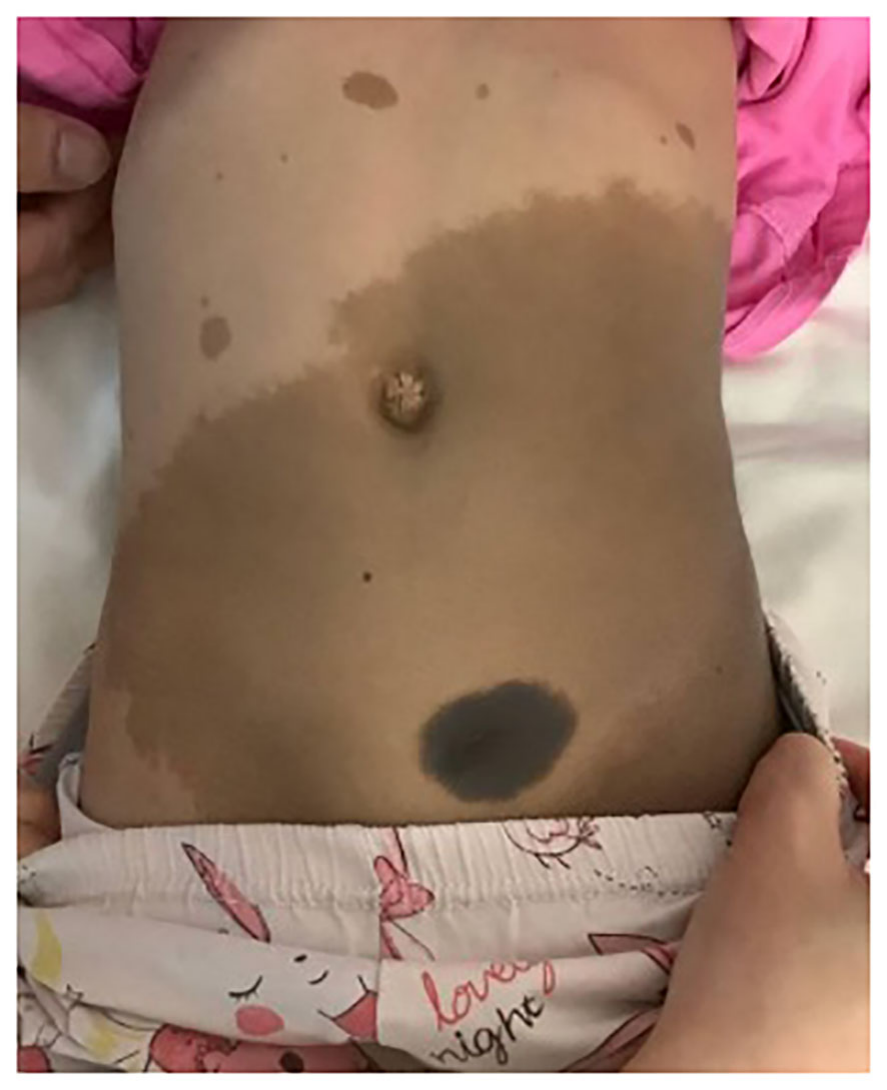
Clinical photo of the index patient demonstrating multiple café-au-lait macules over the trunk and a congenital melanocytic nevus at the lower trunk.

Our index patient is a 4-year-old girl who has been noted to have multiple café-au-lait spots since the neonatal period. She had no family history of neurofibromatosis or multiple café-au-lait macules. A clinical genetics evaluation was arranged for the family at the time, but the clinic visit was not attended by the parents. At 3 years of age, the patient presented with nocturia, urinary frequency up to over 12 times per day, and frequent bowel opening. Physical examination revealed a child with multiple café-au-lait macules over the trunk and back and a congenital hyperpigmented nevus measuring 4 cm × 3 cm over the lower trunk (**Figure 1**), while a pelvic mass was palpable on abdominal examination. Contrast computed tomography demonstrated a heterogeneously and mildly enhancing mass measuring 5.0 cm × 2.7 cm × 8.7 cm at the posterior wall of the urinary bladder. The mass has multiple cystic components and involves the bladder trigone. Both the distal ureters and posterior urethra were closely related to the mass. Magnetic resonance imaging (MRI) of the brain performed showed no abnormality apart from mild thickening of the bilateral maxillary sinus mucosa. Tumor markers including alpha-fetoprotein, beta-human chorionic gonadotrophin, and urine catecholamines were not elevated. A bone marrow examination showed no abnormal infiltration. Blood was taken for exome sequencing, which later identified a heterozygous deleterious variant in the *NF1* gene [c.499_502del p.(Cys167Glnfs*10)], confirming the diagnosis of NF-1.

**Figure 2:**
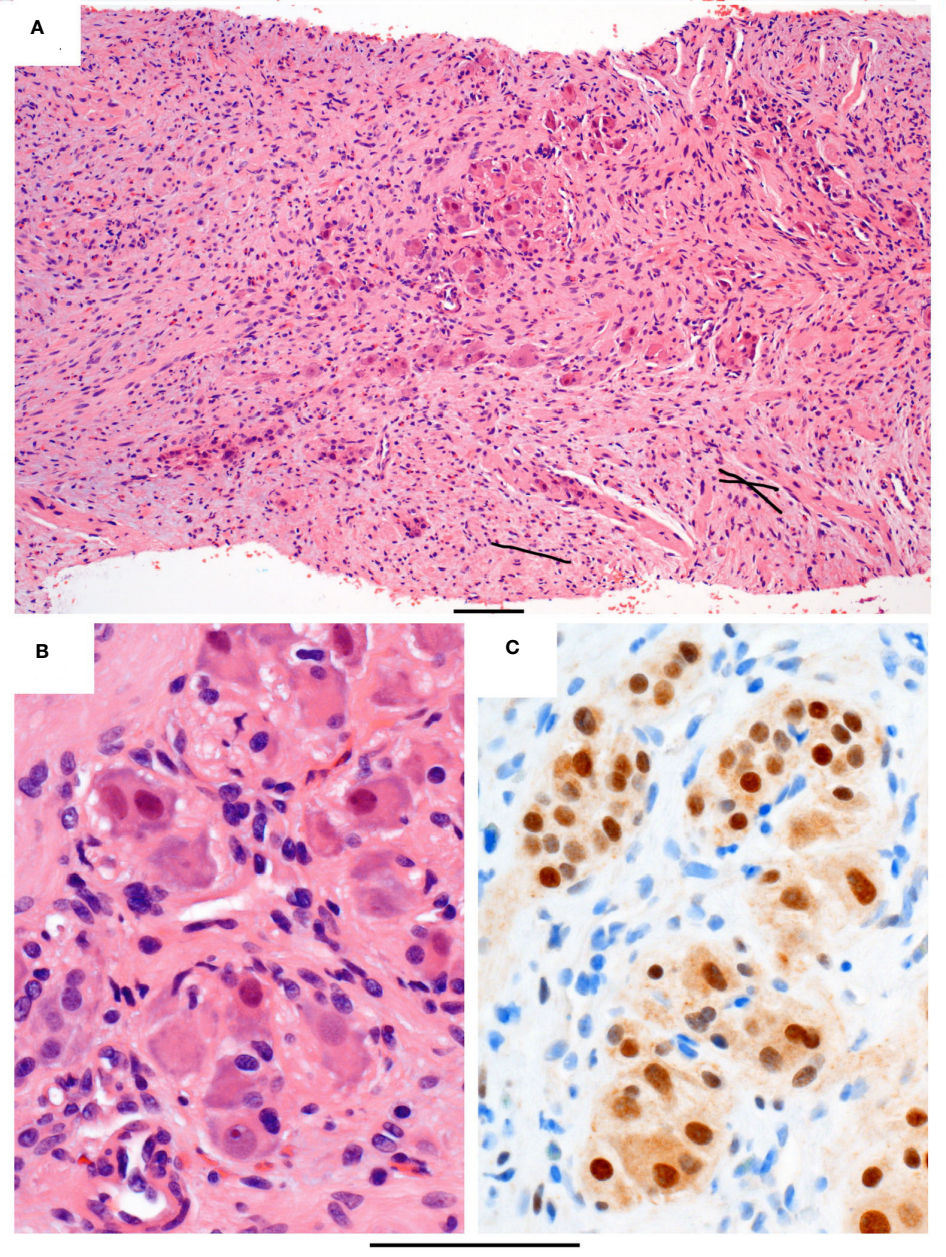
Histopathology of the core biopsy. **(A)** Intermediate power of a representative segment of the biopsy in routine stain showing loose clusters of maturing neuroblastic cells (central area) in a background of spindle Schwannian cells and some inflammatory cells, including eosinophils. **(B)** High power of the maturing neuroblastic cells, including ganglion cells with abundant eosinophilic and peripheral basophilic cytoplasm, round nuclei, and often with prominent nucleoli. **(C)** Immunostain PHOX2B, which is a neuroblastic marker, staining the nuclei of the neuroblastic cells brown. Original magnifications: **(A)** ×100 and **(B, C)** ×400. The bar below the photo indicates 100 µm.

To obtain a tissue diagnosis, a cystoscopy was performed, showing a large reddish growth with cystic areas at the posterior wall, involving the right urinary orifice and trigone. A punch biopsy was taken but was nondiagnostic, yielding only the superficial urothelium and subjacent tissue. An ultrasound-guided core biopsy of the tumor was performed with nine biopsy cores taken, and the pathology revealed predominantly Schwannian spindle cell proliferation in collagenous stroma (**Figure 2A**). There are scattered clusters of mature ganglion cells associated with maturing neuroblastic cells (**Figure 2B**). Immunostaining confirms Schwannian stroma with a strong and diffuse reaction for S100, while the ganglionic and maturing neuroblastic cells are positive for synaptophysin and the neuroblastic marker PhoxB2 (**Figure 2C**). The pathological diagnosis was ganglioneuroma, a maturing subtype.

**Figure 3:**
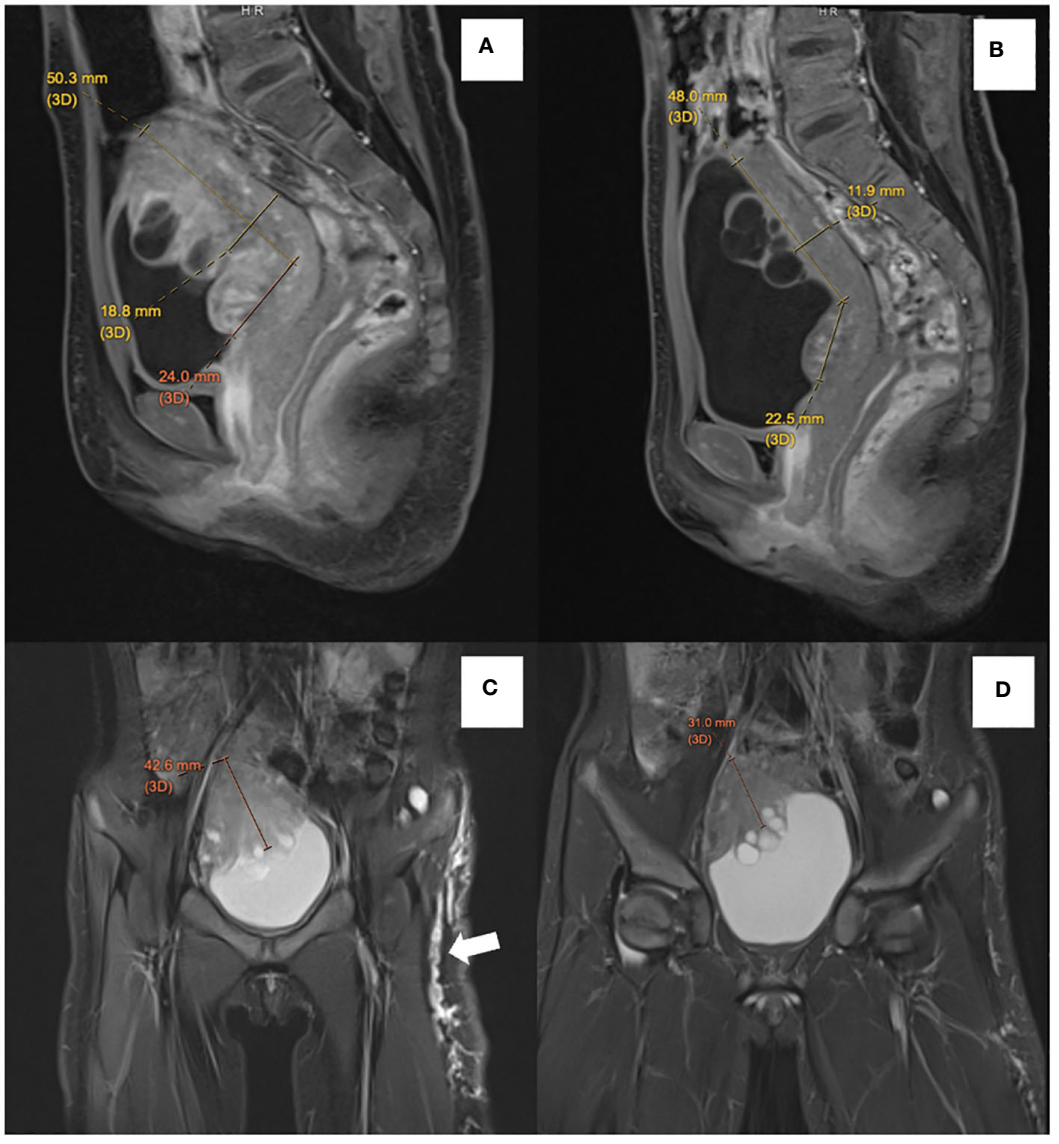
Comparison of sagittal postcontrast T1-weighted images before treatment **(A)** with follow-up MRI **(B)** showed reduced thickness of the solid component of ganglioneuroma from 18.8 mm to 11.9 mm. There is also an interval reduction in the degree of enhancement of solid components. Contrasting coronal T2-weighted short-tau inversion recovery (STIR) before treatment **(C)** with follow-up MRI **(D)** showed a reduction in thickness of the solid component of ganglioneuroma from 42.6 mm to 31.0 mm. Multiple subcutaneous neurofibromata along the left lateral thigh (white arrow) also showed an interval reduction in size and extent in the follow-up MRI.

Due to its involvement in the trigone area, surgery for upfront resection of the tumor was deemed to be unacceptably extensive. As the patient experienced significant urinary and bowel symptoms prior to the availability of genetic confirmation for NF-1, the patient was first started on a mammalian target of rapamycin (mTOR) inhibitor sirolimus, based on *in vitro* studies suggesting treatment response of ganglioneuroma to mTOR inhibitor (11). Nonetheless, the patient had persistent symptoms despite the use of sirolimus. A follow-up MRI of the pelvis performed 6 months after the commencement of sirolimus (**Figures 3A, C**) also indicated a mild interval increase in the size of the main bulk of the posterior bladder tumor (50.3 mm × 42.6 mm × 18.8 mm). The tumor extension toward the trigone measured 24.0 mm. With the diagnosis of NF-1, the patient was switched over to trametinib at the dose of 0.5 mg daily (0.03 mg/kg/day) after a 6-month course of sirolimus. Around 2 months into her trametinib treatment, the patient experienced an improvement in nocturia and had normalization of bowel habits. Interval MRI pelvis showed a gradual reduction in tumor size. Her latest MRI was performed 10 months after initiation of trametinib, showing an interval decrease in tumor extent with less involvement over the left side (**Figures 3B, D**, dimension: 48.0 mm × 31.0 mm × 11.9 mm). The extension toward the trigone also shrank to 22.5 mm. In her latest follow-up, after 15 months of treatment with trametinib, the patient’s urinary frequency has resolved and the bowel opening has normalized. The side effects of trametinib, namely dry skin and alopecia, were tolerable. Physical examination revealed no palpable pelvic mass, and the café-au-lait macules also became fainter in color, while the melanocytic nevus remained static in size and pigmentation.

## Discussion

Ganglioneuromas are benign, slow-growing tumors with low malignant potential. In a retrospective analysis of 328 cases of retroperitoneal, intraabdominal, and pelvic ganglioneuromas, Noh et al. reported that only three cases (1.9%) underwent malignant transformation over a median follow-up of 1.9 years (12). The mainstay treatment of ganglioneuromas is usually surgery. Yet in our case, the tumor abuts the posterior urethra, and the risk of postoperative urinary incontinence and the need for complex reconstruction outweigh the potential therapeutic benefits. Systemic therapy, such as MEK inhibition, became the most feasible approach to management.

The *NF1* gene encodes neurofibromin, which functions as a negative regulator of the RAS protein. Mutation of NF1 results in aberrant activation of the RAS/MAPK pathway, which is central to the tumorigenesis of benign and malignant neoplasms related to NF-1 (13). Inhibitors of MEK1/2, such as selumetinib and trametinib, in turn mitigate the abnormal cell proliferation due to RAS/MAPK signaling. Specifically, MEK inhibitors have become the first class of agents that are FDA-approved for the management of morbidities associated with NF-1 (14).

Recent studies have confirmed the efficacy of MEK inhibitors in the management of NF-1**-**associated plexiform neurofibroma and optic pathway glioma (14–16). Yet little was known about its effect on rarer NF-1**-**related neoplasms, such as those arising from the sympathetic nervous system. This is the first reported case of NF-1**-**related ganglioneuroma showing a clinical and radiological response to the MEK inhibitor trametinib. Interestingly, our patient’s café-au-lait macules, which originate from melanocytes, became fainter after the commencement of trametinib. This may reflect that, just like other cell types originating from the neural crest lineage, trametinib also regulates the cell growth and function of melanocytes.

## Conclusion

This case report illustrates a case of NF-1**-**associated bladder ganglioneuroma, which demonstrated an interval reduction in tumor size and resolution of urinary and bowel symptoms after starting trametinib. Our case has added new understanding to the use of therapeutics targeting the MAPK pathway in the context of NF-1, expanding the applicability of MEK inhibition in these patients.

## Data availability statement

The original contributions presented in the study are included in the article/supplementary material. Further inquiries can be directed to the corresponding author.

## Ethics statement

The studies involving humans were approved by Hong Kong Children’s Hospital Research Ethics Committee. The studies were conducted in accordance with the local legislation and institutional requirements. Written informed consent for participation in this study was provided by the participants’ legal guardians/next of kin. Written informed consent was obtained from the individual(s), and minor(s)’ legal guardian/next of kin, for the publication of any potentially identifiable images or data included in this article.

## Author contributions

MC: Writing – original draft, Writing – review & editing. KF: Writing – review & editing. W-FN: Writing – review & editing. H-ML: Writing – review & editing. DK: Writing – review & editing. AL: Writing – review & editing.
